# Are There Seasonal Variations in Faecal Contamination of Exposure Pathways? An Assessment in a Low–Income Settlement in Uganda

**DOI:** 10.3390/ijerph17176355

**Published:** 2020-09-01

**Authors:** Patrick Ronoh, Claire Furlong, Frank Kansiime, Richard Mugambe, Damir Brdjanovic

**Affiliations:** 1 Environmental Engineering and Water Technology Department, IHE Delft Institute of Water Education, 2611 AX Delft, The Netherlands; c.furlong@un-ihe.org (C.F.); d.brdjanovic@un-ihe.org (D.B.); 2 Department of Environmental Management, College of Agricultural and Environmental Sciences, Makerere University, Kampala 7062, Uganda; fkansiime@gmail.com; 3 Department of Disease Control and Environmental Health, School of Public Health, College of Health Sciences, Makerere University, Kampala 7062, Uganda; rmugambe@musph.ac.ug

**Keywords:** sanitation, SaniPath, seasonal variation, exposure pathways, low-income, Kampala

## Abstract

Sanitation infrastructure are not able to cope with the increasing population in low-income countries, which leaves populations exposed to faecal contamination from multiple pathways. This study evaluated public health risk (using SaniPath) in a low-income community during the dry season, to identify the dominant exposure pathways, and compare this data to existing data for the rainy season, questioning the assumption that risk of faecal contamination is higher in the rainy season. SaniPath was used to collect and assess exposure and environmental data, and to generate risk profiles for each pathway. In the dry season the highest exposure frequency was for bathing and street food, exposure frequency generally increased, and seasonal variation was found in five pathways. The highest hazards in the dry season were through contact with drains, soil, and street food. Seasonal variation was found in the contamination of open drains and street food, with higher levels of *Escherichia coli* (*E. coli*) in the dry season. Open drains were identified as the most dominant risk pathway in both seasons, but risk was higher in the dry season. This highlights the complex nature of seasonal variation of faecal risk, and questions the assumption that risk is higher in the rainy season.

## 1. Introduction

Urbanization, the migration of people from rural areas to cities is one of the crucial demographic trends. According to the latest estimates, the global urban population is expected to grow approximately 1.8% per annum between 2015 and 2020 [1]. The UN predicts that by 2050, the urban population will almost double from 3.3–6.3 billion, with low-income countries bearing the largest increase [2]. In low-income countries the highest increases are found in slums, which have growth rates of 4.5% per annum [3]. It is estimated that about two thirds of the global population live in slums, with the highest prevalence in Sub-Saharan Africa, at 62% [4].

This rapid urbanization and general population growth puts pressure on existing sanitary facilities and many cities are struggling to provide adequate services [5]. The burden is felt mostly in low-income communities including slums, where there is limited planning and facilities. Low-income settlements add a huge burden to city authorities who usually struggle to provide basic services such as sanitation and water. Hence, the immediate surroundings of these populations are highly polluted, and they are therefore prone to enteric diseases due to overcrowding, and inadequate sanitation services [4,6].

In these environments, there are numerous faecal exposure pathways (where a person comes into contact with faecal contamination) that are of public health concern, for example drinking water, public toilets, open drains, street food, and produce [7]. These pathways are usually interconnected and prevalent in the public domain. Hence, sanitation interventions, at household level alone, are not sufficiently capable of reducing the overall exposure to faecal contamination [7]. Several approaches have been designed to identify and quantify the risks faced by populations from these pathways [8]. Exposure and risk assessment tools have been developed and used in urban sanitation to identify and prioritize sanitation interventions based on public health risks [8,9,10].

Most of these tools have only targeted one season [11], which is normally the rainy season [9] or during peak diarrhea season, which is also normally the rainy season [12]. Seasonal variation in risk pathways is complex and poorly understood, but rainfall has emerged as one of the drivers of faecal risk in different regions [13], hence faecal contamination is often detected, and in higher concentrations, during the rainy season [13,14]. This is contextual, as diarrhea cases as a result of faecal risk have been shown to increase during the rainy season [15], while another study found that the level of faecal contamination was lower during the rainy season, compared to the dry season [13]. When seasonality is explored in risk pathways, generally only one risk pathway, normally drinking water sources, is explored [14,16,17], and it is assumed that this pathway is dominant. To date, no studies have explored the seasonality of multiple risk pathways in one community. It is important to know if, and how, the faecal risk from these pathways varies seasonally, so that appropriate interventions can be designed. The SaniPath tool was specifically designed for this purpose, by identifying the frequency and magnitude of exposure to faecal pathways [7].

This study evaluated seasonal variation in public health risks (using SaniPath) from faecal contamination in a low-income community, and identified the most dominant exposure pathways in both seasons, to aid evidence based decision making in terms of sanitation investments.

## 2. Materials and Methods

### 2.1. Study Location

**Figure 1:**
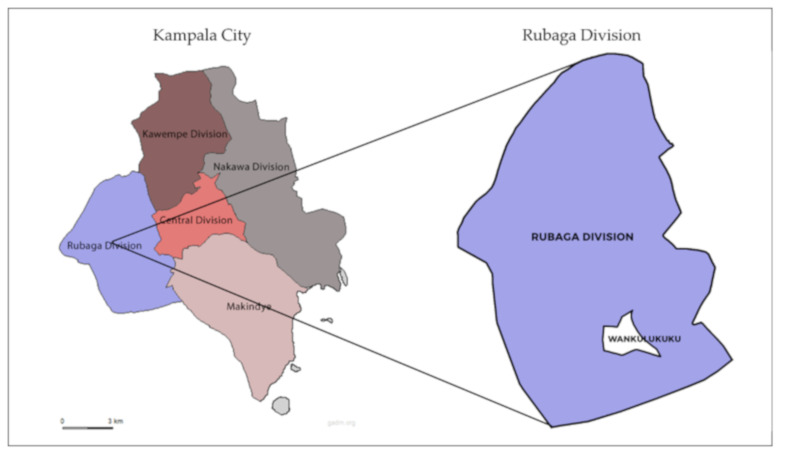
Location of study area.

The study was conducted from January to February 2019 in a low-income settlement, which is defined as a slum by UN-Habitat [3], in Kampala, Uganda. Kampala is Uganda’s capital city and the largest urban, industrial, and commercial center [18] located on the shores of Lake Victoria. It has a population of about 1.5 million people [18], with an urbanization rate of 5.4% per annum and 60% of the city’s residents live in low-income settlements [19]. The city is divided into five administrative divisions: Central, Rubaga, Kawempe, Nakawa, and Makindye (Figure 1), which are further divided into parishes and villages [18]. Kampala has a tropical climate, which features two annual rainy seasons; the first from March to May, peaking in April; and the second from August to December, peaking during October and November (precipitation ranging between 85–153 mm with average temperature of 21.5 °C). The main dry season is from January to February, with a secondary dry season in June and July (precipitation ranging between 64–74 mm with an average temperature of 22.1 °C) [20].

The study was conducted in two villages in a low-income parish in Rubaga division (Figure 1) during the dry season (January to February 2019). Rubaga division is located in the western part of the city. It is divided into 13 parishes with a population of 383,000 people [21]. The low-income settlements [3] in this division are equally distributed between the parishes. The site was pre-selected as this was a comparative case study, the data were compared with a study undertaken during the rainy season (October–December 2018) [22].

### 2.2. Data Collection

This study was part of a larger SaniPath study conducted by Makerere University School of Public Health in collaboration with the Center for Global Safe Water, Sanitation, and Hygiene, Rollins School of Public Health, Emory University. The aim of the overall study was to assess the levels of faecal contamination associated with major faecal exposure pathways, to aid with the prioritization of interventions to control diarrheal diseases.

Both studies, in the rainy and dry seasons, used the SaniPath assessment tool [23]. The SaniPath tool was used because it can quantify the magnitude of faecal contamination in specific exposure pathways, hence it was used to compare and prioritize faecal risk pathways. SaniPath recommends the assessment be undertaken during peak rainy season, to provide a “worst case” scenario [23].

**Figure 2:**
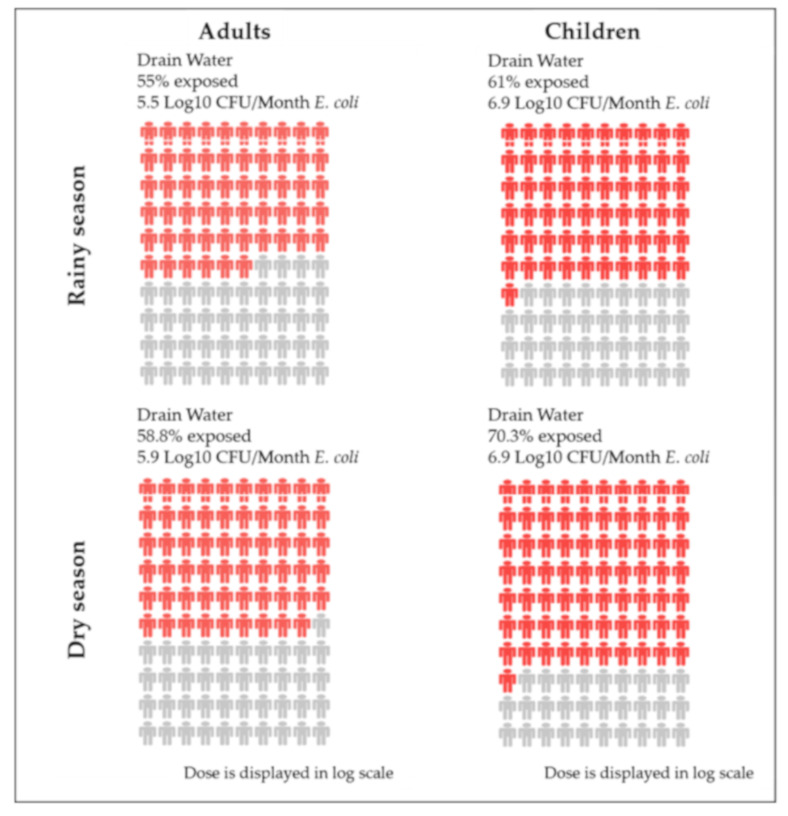
Risk profiles (people plots) for open drains for the rainy and dry seasons. Each red figure on the people plot represents one percent of the population for either an adult or a child that is exposed to faecal contamination in a pathway. The color change of red represents the magnitude of the dose of *Escherichia coli* (*E. coli*) ingested per month, with darker red representing a higher dose. The grey figures represents the population that is not exposed to faecal contamination in a pathway.

The pathways which are specified in SaniPath are drinking water, surface waters, soils, wastewater irrigated produce, bathing water, street food, open drains, flood water, and public latrine surfaces [23]. The tool then automatically generates risk profiles (people plots, Figure 2) for each pathway from the data entered [23]. The people plots of the pathways (Figure 2) illustrate the risk, which can be either through direct ingestion of contamination, through street food, drinking water, raw produce etc. or indirect ingestion via hand to mouth transfer after hand contact with a contaminated surface, like public latrines or open drains and surface waters, and the plots show where interventions will have the greatest impact when it comes to reducing the overall exposure to faecal contamination [7]. The people plots (Figure 2) were generated from data collected on the exposure to faecal contamination gained through the collection of microbiological data on environmental samples, and behavioral data on the frequency of exposure to each environmental sample.

This study used secondary data from a SaniPath assessment, conducted during the rainy season in 2018, and aimed to replicate the study in the dry season in 2019 [22]. The SaniPath methodology details the process of conducting behavioral surveys with community groups and households, and how to collect and analyze environmental samples for *Escherichia coli* (*E. coli*) [23].

The preliminary assessment [22] for the original SaniPath study targeted and identified, the neighborhoods in Kampala that were low–income [3]. Key informant interviews and transect walks, with city officials and community leaders, were undertaken during this preliminary assessment to, identify neighborhoods (Wankulukuku and Suna), identify relevant pathways (street food), and identify environmental sampling sites [22]. These data were then used to develop the sampling strategy for the initial SaniPath assessment, which was undertaken in the rainy season [22].

In the initial SaniPath assessment (during the rainy season) households were randomly selected. In total 100 household surveys were conducted in the initial study [22], 70 of these households were surveyed during the dry season and 30 were new. In this study, two community surveys were conducted with the same participants from the rainy season study [22], and two school surveys were undertaken with the same schools surveyed during the rainy season [22]. The target of the school surveys was children aged between 10 and 12 years, as specified in the methodology [22]. All the surveys contained questions on the frequency with which both adults and children performed behaviors that lead to exposure to faecal contamination from the pathways included in the SaniPath tool.

Environmental samples were collected from the same sites in the public domain as those collected in the rainy season study [22]. The SaniPath tool focuses entirely on exposure to faecal contamination in the public domain because they are areas most affected by sanitation interventions, any contamination in the public domain will likely affect the private domain, and also data collection is more practical in the public areas [24]. Ten samples, which is the minimum recommended number by SaniPath methodology, were collected from each pathway. Samples for each pathway were collected on the same day, as was the sampling strategy in the original study [22]. Before analysis, the samples were kept at 4 °C and all analysis was done within 6 h of taking the sample. The sample types previously identified were: (1) open drain water from an open channel carrying liquid and solid waste, (2) raw produce that is eaten without cooking, (3) drinking water, also referred to as municipal water, supplied by the National Water and Sewerage Corporation, (4) public latrine swabs from walls and handles, (5) street food prepared and sold by vendors on the streets, (6) bathing water commonly used for washing, showering, or bathing in the neighborhood, spring water was identified to be the common type, (7) soil from where children play, and (8) surface water from ponds and swamps (only two samples were collected due to lack of surface water in the dry season) [22]. The samples were collected and processed as per the SaniPath protocols for sample collection and laboratory processing and analyses [23]. Street food samples (from the same vendor and of the same food types as in the previous study) were collected in 2 L Whirl-Pak bags, the vendors were requested to prepare a single serving and place it in the Whirl-Pak bag. In the laboratory, the samples were first mixed well (by compressing them together by hand for 3 min), 10 g of the sample was then homogenised with 90 mL of phosphate buffered saline (PBS) in a sterile 100 mL Whirl-Pak bag for one minute. Swabs from the same latrines as the previous study were collected using EnviroMax Plus sterile environmental sampling swabs. Swabs were taken from latrine walls and door handles and using a T-square, an area of at least 100 cm^2^ (10 cm × 10 cm) on each arm of the T-square was swabbed. In the analysis, 7 mL of phosphate buffered saline with tween (PBST) was added to the swab container and vortexed for 30 s, then incubated for 5 min at room temperature, and vortexed again for another 30 s. The swab elute was poured into an empty 15 mL conical tube. For soil, a sanitized spatula was inserted into the soil at a 45° angle to a depth of 5 cm and a composite of up to seven samples was collected for one soil sample. At the laboratory, 10 g of the soil sample was mixed with 20 mL of PBS and vortexed for 30 s, the pH was then adjusted to 9.0 by adding drops of sodium hydroxide (NaOH), and homogenised with a shaker for 30 min. For produce, the same samples were collected as the previous study (tomatoes and cabbages), and the same procedure was followed as for the street food during collection. At the lab, whole pieces were rinsed with 500 mL of PBST and incubated for 10 min at 37° C. These were then mixed well by vigorously shaking for 30 s, after which the surface of each piece of the produce was gently massaged through the bag for 60 s, and then removed and its weight measured. All the homogenised samples were diluted, as in Table 1, with distilled water. They were then analyzed for *Escherichia coli* (*E. coli*) (an indicator of faecal contamination) using membrane filtration (0.45 µm, 47 mm) and Chromocolt^®^ coliform agar. After filtration, samples were then incubated at 37 °C for 24 h. *E. coli* colonies within the range of 20–200 colony-forming unit (CFU) were enumerated and recorded as described in standard methods 9222 [25]. All microbial concentrations for *E. coli* were log_10_ transformed, and the geometric mean and standard deviation were calculated.

### 2.3. Risk Analysis

The SaniPath tool calculates the risk per month, from the behavioral and environmental data [23]. The dose (number of CFU of *E. coli* ingested per event) was calculated by using the average contamination level (CFU of *E. coli* per unit volume) multiplied by intake (average unit volume ingested per event) [23]. The value for the intake variable was informed by the literature and data from a formative study in Accra, Ghana, and was dependent on the exposure pathway [7]. Dose is one of the major components for calculating the risk, the other component is the average frequency of exposure (number of events per unit time), which comes from the behavioral survey data. One thousand iterations of Monte Carlo simulation were then conducted to estimate, the percent of population that was exposed, dose, and to calculate exposure, with the final product being the people plots for both adults and children (Figure 2) [26].

The dominant pathway(s) of exposure was determined by multiplying the dose and percentage of the population exposed (both generated by the SaniPath tool), which was then log transformed and denoted as *E* [24]. Pathways which have *E* equal or larger than 10, or which fall within a log 1 range of the maximum *E* value, are considered high risk and dominant pathways, if the value of *E* is less than 1 (low risk) for all pathways, then there is no dominant pathways [24].

### 2.4. Statistical Methods

A Wilcoxon signed-rank test (WSR) was conducted to compare the faecal contamination (hazard) and Mann-Whitney U (MW-U) was conducted to compare behavior frequency (exposure), between the rainy and dry seasons. The null hypothesis tested was that there was no difference between the rainy and dry season.

### 2.5. Ethical Approval

This study was a part of a larger SaniPath study which obtained ethical approval from Makerere School of Public Health Institutional Review Board (IRB ethical approval number is 626), which is registered with the Federal Wide Assurance (FWA) number of FWA00011353.). Informed consent was obtained before administering the surveys to the households, community, and schools in the appropriate language (English or Luganda), with a translator where necessary.

## 3. Results and Discussion

### 3.1. Household, Water, Sanitation, and Hygiene Characteristics

It is thought that the reason that the same households could not be surveyed was due to the mobility of the population living in compound housing, respondents living in compound housing decreased from 92–79% [22] from the rainy to dry seasons. Across the two villages, the average household size was five people in both seasons [22]. Hence, the new households interviewed can be assumed to have the same characteristics, in terms of people living in the households, as those who had moved away.

In terms of water and sanitation, all of the respondents reported treating their drinking water (municipal water) either by boiling or chlorination before consumption in both seasons [22]. This was thought to be due to the households’ perception of the quality of tap water, a behavior also observed in households in Kampala in another study [27].

Over half of the respondents (59%) reported sharing the toilet in their compound housing with more than five other households in the rainy season [22], compared to 46% in the dry season. This decrease in shared facilities was probably linked to more people living in compound housing in the rainy, than the dry season. This number of households sharing is more than the recommended four households per toilet stated in Uganda’s National Sanitation Policy [28]. Sharing of latrines is typical in low-income settlements in Kampala, where about 75% of the low-income residents use shared or compound latrines [29].

A majority of respondents (98%) in the rainy season [22], and dry season (92%) reported that their toilet never floods during the rainy season, despite this area being low-lying and susceptible to flooding during a heavy rain event [19]. During the field work it was noted that most of the toilets were elevated and built using plastered brick work, this prevents rain and storm water entering and flooding the systems. This is a common way of constructing toilets in flood prone areas in Kampala, and is also used due to the high water table in this area (<1.5 m below the surface) [29,30,31].

### 3.2. Environmental Contamination

Table 2 shows the *E. coli* geometric mean for five pathways for the rainy [22] and dry seasons and the *p*-Values for the WSR.

#### 3.2.1. Open Drains

The detection of high numbers of *E. coli* in all the samples in both the dry and rainy season is an indication of inadequate faecal sludge management in this community. This was evident as latrines were discharging faecal sludge directly into drains in this community, which is a common practice in Kampala [29,30]. The levels of contamination found in the dry season are similar to those found in a quantitative microbial risk assessment (QMRA) study undertaken in the dry season in Kampala (6.9 log_10_ CFU per 100 mL) [32]. Open drains had the highest levels of *E. coli* contamination in both seasons (Table 3). As expected, the geometric mean concentration of *E. coli* was lower and less variable in the rainy season compared to the dry season (Table 2). This was due to the absence of dilution through rainwater. This seasonal variation was found to be significantly different (*p* ≤ 0.05, Table 2). The only other study that explored seasonal variation in *E. coli* concentration in open drains was from Ghana, though they did not find any variations, and the study looked at two rainy seasons [33]. The results are surprising because it would have been expected that the risk would have been higher during the rainy season than the dry season because it has been observed that most elevated latrines in Kampala have an opening at the back which is used to empty faecal sludge into adjacent open drains, in addition to the washing effect of rain [30]

#### 3.2.2. Soil

Soil had the second highest level of *E. coli* contamination in both seasons (Table 2). The geometric mean concentration level of *E. coli* in soil was higher in the rainy season than the dry season (Table 3), similar to a study in Bangladesh [34]. The higher concentration detected during the rainy season was possibly due to the increased moisture in the soil which creates a conducive environment for microorganisms such as *E. coli,* leading to longer survival [35]. This brings into question of the use of *E. coli* as an indicator for tropical climates [36]. Other activities such as discharge of faecal waste during the rainy season can also lead to the contamination of soil. A study in an urban slum in Uganda found higher levels of *E. coli* (5.4 log10 CFU per g), compared to this study during the dry season, but there, samples were taken specifically from open spaces and the playgrounds of children [33]. Children face considerable risk from soil during the dry season, this is because they spend most of the time playing outside on the ground.

#### 3.2.3. Compound Latrines

As there were no public latrines in these villages, samples were taken from compound shared latrines, as in the original study [22]. These latrines had an average of two toilets/stances per block with a median of 50 users per day. From the observation during swabbing in the dry season, feces was visible on the walls and/or the slab in all ten latrines. In addition, none of the latrines in either season had handwashing stations nearby, a common observation in Kampala [30]. Despite the walls having feces, only one latrine swab in the dry season was positive for *E. coli* with a concentration of 3.82 log_10_ CFU per swab, while in the rainy season no swab was positive for *E. coli* [22]. This is surprising because a study in Kampala found compound latrines in the rainy season to be very dirty (presence of urine, solid faecal matter, dirty paper, or any other refuse on the slab or squat hole), unlike the dry season where they were much cleaner, therefore seasonal variation was expected [37].

A failure to detect *E. coli* from the latrines swabs in both seasons could be attributed to a number of factors including, the type of swab used [38], the recovery rate from the swabs [38], and the type of surface sampled [38]. For this particular study, swabbing was only conducted on the door handles and the walls (which people touch), this could have affected the amount of *E. coli* detected.

#### 3.2.4. Raw Produce

The geometric mean concentration of *E. coli* detected in the raw produce was higher in the rainy season than in the dry season (Table 2). The detection of *E. coli* in the raw produce in both seasons indicates a recent faecal contamination since the crops were not grown locally nor irrigated by wastewater. This post-harvest contamination happened during handling and storage of the produce by the vendors. Hence, the contamination during the dry season was thought to be due to the produce being stored on the ground. The higher levels of contamination in the rainy season were thought to be due to the muddy environment and the increase in moisture, leading to longer *E. coli* survival [35]. Similar findings were reported in Ghana where higher levels of faecal contamination were detected in lettuce during the rainy season than in the dry season, although not significantly different [39]. In this study the seasonal variation was not found to be significantly different (*p* ≥ 0.05, Table 2).

#### 3.2.5. Bathing Water (Spring Water)

Spring water was used by a minority of respondents for bathing (32% during the rainy season [22] and 22% during the dry season), and was analyzed for *E. coli* contamination (Table 2). The contamination of unprotected springs during the dry season is similar to a QMRA in Kampala (2.5 log10 CFU per 100 mL) [33]. The geometric mean concentration level of *E. coli* in springs in the rainy season was slightly lower compared to the dry season (Table 2). Slightly higher levels of contamination in the dry season could be as a result of a decrease in the water levels in the unprotected springs. Contamination of unprotected springs is attributed to inadequate and poor sanitation facilities within the neighborhood, which at times can lead to open defecation, and unsafe disposal of faecal sludge [40]. In terms of seasonal variation, a similar pattern was observed in Ghana, during the wet season the level of concentration ranged from 1.5 to 3.10 log_10_ CFU per 100 mL, and that of the dry season ranged from 2.0–3.08 log_10_ CFU per 100 mL, with no significant difference between the wet and the dry seasons [41]. Despite higher *E. coli* levels during the dry season, there was no significant seasonal variation (*p* ≥ 0.05, Table 2).

#### 3.2.6. Drinking Water

Drinking water in both seasons had no detectable *E. coli* contamination [22]. This implies that the water met WHO recommended guidelines for drinking water [42]. A review of seasonal variation of faecal contamination in improved drinking water sources in developing countries found a seasonal trend of higher faecal contamination during the rainy season [14], which this study did not find.

#### 3.2.7. Street Food

Street food in the rainy season had less faecal contamination with only one sample having *E. coli* of 2.57 log_10_ CFU per g, all other samples had too few to count *E. coli* counts, whereas in the dry season all ten samples had countable *E. coli* with a geometric mean of 3.55 log_10_ CFU per g (SD: 0.49 log_10_ CFU per g). The street foods sampled were rolex, kikomando, and samosa (described in Table 4). Consistent levels of contamination during the dry season can be attributed to dust and the uncovering of food and utensils. In addition, lack of sufficient water during the dry season for hygienic activities, like cleaning of surfaces, handwashing, and washing of utensils, could be another reason for higher concentration compared to the rainy season. This seasonal variation was found to be significantly different (*p* ≤ 0.05, Table 2). No earlier studies looked at seasonal variation and the contamination of street food, hence this warrants more exploration.

### 3.3. Behaviour Frequency

Table 1 shows a summary of behavior frequency of the eight pathways for adults and children in the rainy and dry seasons. For one pathway (soil), this information was not captured because the SaniPath tool does not have a behavioral question for it.

#### 3.3.1. Bathing Water

Bathing water had higher exposure than any other pathway in both seasons, more than 90% of adults and 70% of children were exposed to it more than ten times in a week (Table 3). High exposure frequency was because each respondent reported to shower at least once in a day, and the desire to be clean made people bathe more often so as to appear tidy, as seen in other places [43]. During the rainy season, the water used for bathing was either from a tap (supplied by National Water and Sewerage Corporation, NWSC), which was used by 68% of the respondents, or spring water, used by 32% of the respondents [22], while in the dry season, national water was used by 58% of the respondents, spring water used by 22%, or both NWSC and spring water used by 20% of the respondents. No significant seasonal variation was found in exposure to bathing water in either adults or children (*p* ≥ 0.05, Table 3).

#### 3.3.2. Drinking Water

Drinking water had the second highest exposure in both seasons (Table 3), 60% of children and adults reported drinking water supplied by NWSC every day during the rainy season [22], compared to over 70% in the dry season for both adults and children (Table 3). Other sources of drinking water that were used included rain, bottled, and spring water. Despite no detectable *E. coli* contamination in these studies (Table 2), all of the respondents in both seasons [22] still treated their water, either by boiling or chlorination, before drinking, this is an indication that households do not perceive tap water as being of good quality for drinking. The treatment of household water in this context will not affect the calculation of risk by the SaniPath tool, but if the drinking water was of lower quality and this practice was ignored by the SaniPath tool the risk from this pathway would be overestimated. Consumption of municipal water every day was probably due to the availability, as households could easily access public standpipes and water kiosks. No significant seasonal variation was found in exposure to drinking water (municipal water) for both adults and children (*p* ≥ 0.05, Table 3).

#### 3.3.3. Street Food

Street food, which includes rolex, kikomando and samosa (Table 4), had a lower exposure in the rainy season compared to the dry season, 21% of adults and 39% of children consumed street food more than ten times in a week during the rainy season [22], but this increased to 66% of children and 48% of adults in the dry season (Table 3). In both seasons, the consumption of street food was higher in children, similar to a study in Mali [44].

Rainy seasons are characterized by an abundance of agricultural produce, hence a wider variety to choose from unlike the dry season, this could explain why less street food was eaten in a week in the rainy season. These foods are usually prepared and sold from stands on the street due to their low prices (0.24 euros for rolex and kikomando, and 0.12 euros for samosa), limited time to prepare food, changing lifestyles, and accessibility, street foods provide an accessible source of food and employment to people living in low-income settlements. Apart from the relatively cheap prices and instant access, street food has also become an essential component in maintaining the nutritional status of low-income dwellers, as it contributes significantly to the amount of protein and daily energy for both adults and children [44]. In terms of seasonal variation, this is the first study to explore this. This seasonal variation in consumption of street food was found to be significant for both children and adults (*p* ≤ 0.05, Table 3).

#### 3.3.4. Open Drains

There was also a considerable amount of exposure to open drains in both seasons (Table 3). Exposure to open drains was highest for children while it reduced for adults in the dry season (Table 3). In the dry season the proportion of children increased, with 43% of the children coming into contact with open drains more than ten times in a month, while that of adults slightly reduced to 33% (Table 3). The lower exposure frequency of children to open drains during the rainy season was possibly due to the environment being unfavorable for children to play outside, and full drains being perceived as dangerous, meaning that children spent time indoors during the rainy season. The opposite may be true in the dry season, leading to a higher frequency of exposure of children in this season. In addition, all the drains did not have covers and this could also lead to high exposure for children, similar to a SaniPath study in Ghana [45]. Although the adults’ exposure to open drains was not found to be significantly different between seasons (*p* ≥ 0.05, Table 3), children’s exposure was found to be significantly different between seasons (*p* ≤ 0.05, Table 3). As significantly higher amounts of *E. coli* were found in samples during the dry season, these findings (Section 3.2.1) may have significant consequences for children in this community. Previous SaniPath studies have focused on exposure during the rainy season, therefore the risk from contact with open drains may be underestimated.

#### 3.3.5. Raw Produce

In the rainy season, consumption of raw produce, one to five times a week, in both adults and children was almost identical at 38% and 39% [22], respectively (Table 3). In the dry season, the proportion of consumption of produce, one to five times, was 28% for adults and 26% for children. The weekly consumption of raw produce decreased from the rainy to the dry season (Table 3), but it should be noted that generally raw produce consumption is lower than the consumption of street food (Table 3), probably because consumption of raw produce usually happens as part of salads, and in this particular area, salads are not a major part of the diet, and this explains the low frequency of exposure to raw produce. The differences in consumption of raw produce could be due to the seasonality of the produce, with more being available during the rainy season, which leads to cheaper prices. This seasonal variation in consumption of raw produce was found to be significantly different for both children and adults (*p* ≤ 0.05, Table 3).

#### 3.3.6. Public Latrines

Public latrines had one of the least exposures for both adults and children in the dry season. In the rainy season, 48% of adults and 33% of children reported never using public latrines in a week, while 21% of adults and 34% of children used them more than ten times in a week (Table 1) [22]. In the dry season, 67% of children and 62% of adults reported never using public latrines in a week, while 23% of adults and 22% of children reported using the public latrines more than ten times in a week (Table 3). Generally, the low exposure to public latrines in both seasons was because most respondents had toilets in their compounds, which they shared with other households, and those who reported using public latrines, did so when they were out of their community. Toilet ownership is not the only reason that could prevent people from using public toilets, cleanliness is another major concern. In a study in slums in Kampala, Uganda, respondents cited lack of cleanliness as a reason for not using public toilets, this is in addition to the high traffic of users, and the cost for using the public latrines [46,47]. The exposure to public latrines was higher during the rainy season and this seasonal variation in exposure was significantly different for both adults and children (*p* ≤ 0.05, Table 3).

#### 3.3.7. Surface Water

People in this study had the lowest exposure to surface water compared to all other pathways (Table 3). In the rainy season 80% of children and 86% of adults [22] reported having never come into contact with surface water in a month (Table 3). During the dry season, 68% of children and 82% of adults reported having never come into contact with surface water in a month (Table 3). The likely reason for low exposure to surface water is that Rubaga division has no lakes or rivers, and in addition the swamps available in the division are mostly seasonal. As expected the seasonal variation in exposure to surface water (which is seasonal) was found to be significantly different for both children and adults (*p* ≤ 0.05, Table 3).

#### 3.3.8. Seasonal Variation in Behavior

Drinking water, which is usually the focus of most seasonality studies, was found to have no significant difference in terms of behavior in the rainy and dry seasons for both adults and children. In addition, there was also no change in behavior in the exposure to bathing water, in this case spring water. For surface water, the difference in exposure is related to the amount in the environment, with higher amount in the rainy season. Open drains are the only pathway that had a difference in seasonality between children and adults, with significant difference found in children’s exposure to open drains, which is related to the amount of time they spent outside, close to the drains, in both seasons. The consumption of street food and raw produce was found to have seasonality, this is interesting because they are some of the neglected pathways when in it comes to water, sanitation, and hygiene (WASH). In terms of public latrine usage, seasonal variation was found, in this context seasonal variation is in how they use the outside space. Little seasonal variation was found in WASH practices, with difference in exposure for children and adults to open drains.

### 3.4. Risk Profiles and People Plots

Table 5 shows the percentage of adults and children exposed, dose per month, and *E* scores (risk) for seven pathways generated by the SaniPath tool. From these pathways, open drains and raw produce had valid risk profiles for both seasons, street food had valid risk profiles for only the dry season, while bathing water and drinking water had flawed risk profiles for both seasons.

From Table 5 it appears as if bathing water is a major pathway due to 100% exposure and relatively high doses per month, but the risk profile for bathing water was flawed. This was because a majority of respondents in both seasons used municipal piped water (drinking water) (Table 3) for bathing which had no detectable *E. coli* contamination in both seasons (Table 2). The SaniPath tool based its analysis on spring water samples (Table 2) and therefore the risk in terms of dose is overestimated.

Street food risk profiles for the rainy season were not valid, as the SaniPath tool required ten valid environmental results to generate valid risk profiles, but only six samples for street food were valid for the rainy season. With a lower sample number, more bias is introduced, meaning the dose calculations are less accurate.

For drinking water, Table 5 shows that adults and children in both seasons were exposed to some level of dose, however it is unclear how the doses were calculated because no faecal contamination was detected in drinking water in either season (Table 2). Additionally, respondents treated their water in both seasons before consumption. This means that there is an overestimation of risk for this pathway.

This study (dry season) was constrained, as it was replicating the study undertaken in the rainy season [22]. It was found that in implementing a SaniPath study, a good understanding of how behavior and contamination are linked is required, to avoid gaining flawed overall results, e.g., the cases of bathing practices and spring water. Additionally, a high level analysis is required when selecting the dominant pathways and interpreting the results, e.g., the case of drinking water (Table 5). In addition, the correct number of samples need to be obtained (10 samples per pathway as recommended by SaniPath), to avoid having flawed results, for example in the case of street food in the rainy season. Hence, when implementing a SaniPath study an in-depth understanding of both the tool and context is required.

### 3.5. Most Dominant Pathways

From Table 5 it can be seen that *E* scores range from 0–8.4, a dominant pathway is a pathway with an *E* score of ≥10 or 1 log range from the maximum *E* score [26]. The dominant pathway was only present during the rainy season (flood water, Table 5), as this study was exploring seasonal variation in pathways, a pathway was considered dominant if the *E* score ranged from 5.8–6.8, as 6.8 was the maximum *E* score for a pathway which is present in both seasons (open drains, Table 5).

Interestingly it can be seen that the pathways in Table 5 that are traditionally linked to WASH (drinking water, bathing water, and latrine contact, although it should be noted the pathways had flawed sampling strategies) have low *E* scores, and the dominant pathways are not traditionally seen as WASH pathways. This highlights the importance of exploring beyond traditional WASH pathways, as they pose the greatest public health risk if contaminated with faecal matter.

#### 3.5.1. Flood Water

A dominant pathway occurring only in the rainy season was flood water (Table 5). The proportion of adults and children exposed to it was less compared to the other pathways, but it recorded one of the highest doses for both adults and children (Table 5). The *E* scores for both adults and children were more than 8 (Table 5). Contamination in flood water usually happens as a result of runoff, whereby the rain water washes all the contaminants into the environment, and re-distributes and remobilizes sediments, which can contain faecal matter as a result of open defecation or unsafe disposal of waste [48].

#### 3.5.2. Open Drains

Open drains is dominant only in children in both seasons, but only a dominant pathway for adults in the dry season (*E* scores > 5.8, Table 5). The risk profile (people plots) for open drains is depicted in Figure 2, it can be clearly seen that the risk is higher in the dry season (Table 5) compared to the rainy season, due to the number of those exposed (supports the findings in Section 3.3.4) and the level of contamination (supported by the results in Table 3). The people plots show a visual comparison of exposure across the pathways, seasons, and population (adults or children), and can be used to provide feedback to communities. In both seasons, the risk was higher in children than in adults, with more children getting exposed, and to higher doses (Section 3.3.4). The risk from open drains is similar to a QMRA conducted in Kampala where exposure to open drains contributed to the highest disease burden (39%) [32]. This is surprising as it is normally assumed that risks are higher in the rainy season compared to the dry season, which means the risk of children playing in open drains is underestimated in the dry season.

#### 3.5.3. Street Food

Street food is dominant for both adults and children in both seasons (*E* scores >5.8, Table 5). Although it should be noted that, during the rainy season the minimum number of samples were not collected (Table 2). In addition, it has the highest proportions of both adults and children exposed to it in both seasons (Table 5). The proportion of children consuming street food was higher than for adults, but the doses were lower (Table 5). This difference in dose is likely because of the portion of street food consumed per serving. Adults basically consume a larger portion of food compared to children, because of the amount of energy they need [44].

### 3.6. Recommendations

In terms of interventions, the city authorities should focus on sanitation investments that will tackle exposure to the dominant pathways; street food (dominant in the dry season), open drains (dominant in both seasons), and flood water (dominant in the rainy season).

For street food, strengthening of policies, formulation of guidelines, and proper enforcement would greatly reduce the risk posed by street food and ensure its safety [49]. To achieve all these, it is necessarily to involve all the stakeholders in the street food vending business, such as the street vendors, consumers, national and local governments, and civil societies. Public education is another aspect that can be applied in trying to reduce the risk from street food [50]. For all the stakeholders involved, awareness of the dangers posed by unhygienic handling of street food can be disseminated to them through the mass media. For the street food vendors, they can be trained in food safety at all stages of the production chain. While regulation and education are important for street food safety, not taking into consideration the physical infrastructure might be detrimental to the public health initiatives. The local governments should come into play by providing adequate infrastructure with services, for example vending stalls, clean water supply, sanitary facilities, as well as waste disposal facilities. The provision of sanitary facilities and clean water is crucial when it comes to reducing the food borne diseases that result from street food consumption [51].

In terms of reducing risks posed by open drains, the best intervention is that aimed at preventing direct contact between the population and the contaminated open drain water [52]. This will involve physical and engineering approaches in the slums. One approach is through the provision of covers which can be easily removed to facilitate operation and maintenance of the drains [52]. Open drains in low-income settlements are also used as solid waste disposal sites. The solid waste dumped in them end up clogging the drains, which reduces their flow capacity. The local government can provide solid waste disposal facilities in this slum. Another possible solution is through installing solid waste traps at strategic locations along the open drains. Solid waste traps have been used to collect and remove solid waste from a flowing drain [53].

The success of these interventions depend on one important element, community engagement [54]. It is important to take into consideration the views of the people the intervention intends to help, as this may have a negative impact on the interventions.

## 4. Conclusions

Surprisingly, exposure frequency between the two seasons was significantly different for most of the pathways (open drains, street food, public latrines, raw produce, and surface water), therefore it is clear that the behavior of people in this particular low-income community is affected by season. In terms of contamination of the exposure pathways, there were higher contamination levels detected in open drains and street food during the dry season. Therefore, there is a probability of underestimation of these risks in this pathway if SaniPath assessment is only undertaken during the rainy season. In addition, when implementing a SaniPath study, an in-depth understanding of both the tool and context is required, as this led to the shortcomings in the original study, some of which were replicated in this study. This is an important finding for those planning a SaniPath assessment. Even with the limitations of the results gained, the SaniPath tool was used to assess public health risks as a result of poor sanitation and to suggest evidence based interventions. What was surprising was that the dominant pathways identified were all non-traditional WASH pathways e.g., open drains, street food, and floodwater. The results of this study will contribute to the evidence base that is available to sanitation policy makers and implementers in low-income and informal urban areas, in the case of this study, Kampala Capital City Authority (KCCA).

## Figures and Tables

**Table 1:** Recommended dilutions for sample type.

Sample Type	No Dilution	Dilution 1	Dilution 2	Dilution 3	Dilution 4
Drinking Water (Municipal Water)	✔	-	-	-	-
Bathing Water (Spring Water)	✔	-	-	-	-
Drain Water	-	1:10	1:100	1:1000	1:10,000
Produce	-	1:10	-	-	-
Street Food	-	1:10	-	-	-
Latrine Swabs	-	1:10	-	-	-
Soil	-	1:10	1:100	-	-

**Table 2:** *E. coli* geometric mean and Wilcoxon signed-rank (WSR) test.

	*E. coli* Geometric Mean					WSR
**Pathway**	**Unit**	**Rainy [22]**	**SD**	**Dry**	**SD**	***p*-Value**
Open Drains	Log_10_CFU/100 mL	6.52 (*n =* 7)	0.17	6.80 (*n =* 10)	0.38	0.010
Soil	Log_10_CFU/g	3.79 (*n =* 6)	0.27	3.65 (*n =* 10)	0.36	0.419
Produce	Log_10_CFU/g	3.26 (*n =* 7)	0.93	2.38 (*n =* 10)	0.03	0.371
Bathing Water(Spring Water)	Log_10_CFU/100 mL	2.81 (*n =* 9)	0.20	2.84 (*n =* 10)	0.39	0.100
Street Food	Log_10_CFU/g	2.57 (*n =* 6)	0.00	3.55 (*n =* 10)	0.49	0.002
Drinking Water(Municipal Water)	Log_10_CFU/100 mL	TFTC * (*n =* 10*)*	-	TFTC * (*n =* 10)	-	-

* TFTC = too few to count <20 CFU/100 mL.

**Table 3:** Behavior frequency for the eight pathways for adults and children in the rainy [22] and dry seasons and the *p*-Values for the Mann-Whitney U(MW-U).

		Rainy Season [22]	Dry Season			*MW-U*
		Adults%	Children%	Adults%	Children%	
**Pathway**	**Frequency of Exposure**	%	%	%	%	*p*-Value
**Surface Water**	More than 10 times in the past month	8	4	2	12	adults *p* = 0.002
	6–10 times in the past month	4	2	2	5	children *p* = 0.016
	5 times or less in the past month	7	6	12	14	-
	Never	80	86	82	68	-
	Do not know	1	2	2	1	-
**Open Drains**	More than 10 times in the past month	36	25	32	43	adults *p =* 0.075
	6–10 times in the past month	36	8	7	10	children *p =* 0.001
	5 times or less in the past month	12	20	10	15	-
	Never	44	39	43	32	-
	Do not know	0	8	1	0	-
Drinking Water *	Every day	61	60	72	76	adults *p* = 0.109
	4–6 days within the past week	3	8	9	12	children *p* = 0.074
	3 days or less within the past week	5	9	5	1	-
	Never	29	22	12	11	-
	Do not know	2	1	2	0	-
**Bathing Water (Spring Water)**	More than 10 times in the past week	91	70	93	71	adults *p* = 0.236
	6–10 times in the past week	7	28	4	26	children *p* = 0.865
	5 times or less in the past week	0	1	1	2	-
	Never within the past week	0	0	0	0	-
	Do not know	2	1	2	1	-
**Raw Produce**	More than 10 times in the past week	10	8	13	15	adults *p* = 0.040
	6–10 times in the past week	13	10	20	23	children *p* = 0.001
	5 times or less in the past week	38	39	28	26	-
	Never within the past week	34	41	34	35	-
	Do not know	5	2	5	1	-
**Street Food**	More than 10 times in the past week	21	39	48	66	adults *p* = 0.000
	6–10 times in the past week	37	34	13	11	children *p* = 0.000
	5 times or less in the past week	29	21	15	18	-
	Never within the past week	11	5	18	4	-
	Do not know	2	1	6	1	-
**Public Latrines**	More than 10 times in the past week	21	34	24	22	adults *p* = 0.006
	6–10 times in the past week	19	15	3	2	children *p* = 0.000
	5 times or less in the past week	10	8	6	8	-
	Never within the past week	48	33	62	67	-
	Do not know	2	10	5	1	-

* This is also referred as municipal water supplied by National Water and Sewerage Company.

**Table 4:** Street food type and its preparation.

Food type	Ingredients	Cooking Method	Serving
Rolex	Eggs, chapati, onions, cabbage and tomatoes	The chapati is usually prepared in advance, it is a flat bread made from wheat flour fried on a pan. The onions, tomatoes, and cabbage are mixed with one egg and fried to make an omelette. The omelette is then wrapped in the chapati	Chopped on a board and served by hands. Usually put in a polythene bag
Samosa	Wheat flour, rice and minced meat	The wheat flour is used to make a shell which is stuffed with either minced meat or rice and then deep fried	Packaged into a polythene bag by hands
Kikomando	Chapati and beans	Boiled beans served with a chapati	Beans served with a spoon in plate, and chapati is first chopped and served by hand.

**Table 5:** Risk profiles generated by the SaniPath tool, showing percentage of adults and children exposed, dose per month (Log_10_ CFU/Month *E. coli),* and *E,* which represents overall risk [26].

Rainy Season [22]							Dry Season					
	**Adults**			**Children**			**Adults**			**Children**		
**Pathway**	**Exposure**	**Dose (Log_10_ CFU/month)**	***E* (Risk)**	**Exposure**	**Dose (Log_10_ CFU/month)**	***E* (Risk)**	**Exposure**	**Dose (Log_10_ CFU/month)**	***E* (Risk)**	**Exposure**	**Dose (Log_10_ CFU/month)**	***E* (Risk)**
Open Drains	55%	5.5	5.2	61%	6.9	6.6	58.8%	5.9	5.7	70.3%	6.9	6.8
Street Food *	93%	6.2 *	6.2	98%	5.8 *	5.8	83.9%	6.9	6.8	96.6%	6.5	6.4
Raw Produce	67%	5.6	5.4	60%	5.4	5.2	66.9%	3.4	3.2	69.6%	3	2.8
Bathing Water **	100%	4.4 **	4.4	100%	5.2 **	5.2	100%	5.5 **	5.5	100%	5.5 **	5.5
Drinking Water **	73%	2.1 **	1.9	78	1.7 **	1.5	90.7%	2.4 **	2.3	91.1%	2 **	1.9
Public/Shared Latrines **	53% **	0.3	0.0	64% **	0.6	0.4	32.3% **	0.5	0.0	32.9% **	0.4	0.0
Flood Water	40%	8.8	8.4	46%	8.8	8.4						

* Number of samples required to calculate the risk profiles were less than the recommended. ** Flawed sampling strategy.
